# Eosinophilic interstitial nephritis and cardiac insufficiency in Kimura’s disease: a case report

**DOI:** 10.1186/s12882-021-02454-3

**Published:** 2021-06-30

**Authors:** Li Xiang, Hua Zhou, Hua Liu, Dachuan Zhang, Min Li, Min Yang, Yan Yang

**Affiliations:** 1 Department of Nephrology, The Third Affiliated Hospital of Soochow University, No. 185, Juqian Road, Changzhou, 213003 Jiangsu Province China; 2 Department of Pathology, The Third Affiliated Hospital of Soochow University, Changzhou, 213003 China

**Keywords:** Kimura’s disease, Eosinophilic interstitial nephritis, Cardiac insufficiency

## Abstract

**Background:**

Kimura**’**s disease (KD) is a rare chronic inflammatory disease and the etiology remains uncharacterized. The typical manifestations are painless lymph node or subcutaneous masses. There is currently no report of prominent renal interstitial injury and cardiac insufficiency in KD.

**Case presentation:**

A 45-year-old man was referred to our hospital with dark urine, subcutaneous masses in forehead and right retroauricular, multiple lymphadenopathy and unexplained cardiac insufficiency. Renal biopsy demonstrated eosinophilic interstitial nephritis. Laboratory tests revealed eosinophilia and a high level of serum IgE. A biopsy of cervical lymph node was performed and KD was diagnosed. Treatment with oral prednisone resulted in a decrease of eosinophil, serum IgE, improvement of cardiac function, and regression of the subcutaneous mass.

**Conclusions:**

We describe an extremely rare KD case presenting with eosinophilic interstitial nephritis, cardiac insufficiency and significant response to prednisone. The clinicians should improve the disease awareness and find optimal treatment.

## Background

Kimura’s disease (KD) is a rare chronic inflammatory disease and the etiology remain uncharacterized. KD was first reported in a Chinese study by Kim in 1937 [1] and was described and named by Kimura in 1948 [2]. It usually manifests as painless subcutaneous masses, regional lymph nodes enlargement, and occasional involvement of kidney. Proteinuria was found in 12–16% KD patients [3], of which 62% KD presented with nephrotic syndrome [4]. There are extremely rare reports on KD with cardiac damage [5]. There is currently no report of prominent renal interstitial injury and cardiac insufficiency in KD. Thus, we report a KD presenting with kidney involvement earlier than subcutaneous mass that renal biopsy showed tubulointerstitial injury and also accompanied with cardiac insufficiency.

## Case presentation

A 45-year-old man presented with dark urine that had lasted for 3 months in September 2019. The main clinical manifestations were hematuria, backache and occasional activity chest tightness. He presented no symptoms related to urinary irritation. Back in December 2010, the patient had symptoms of lower extremity edema and heavy proteinuria with 24-h urinary protein of 20.6 g and serum albumin of 15.4 g/L, and was diagnosed with “nephrotic syndrome”. The white blood cell (WBC) count was 14 **×** 10^9^/L, with 34% eosinophils. He had good response to corticosteroid therapy. After 1 week of prednisone treatment, urinary protein decreased to 1.02 g/day, and eosinophil decreased to 2.19%. However, the patient experienced frequent relapse of nephrotic syndrome and eosinophilia, when prednisone was reduced to 10 mg/day. He repeatedly refused renal biopsy. Additional use of tripterygium wilfordii was ineffective. Proteinuria decreased rapidly in 1**–**2 weeks when prednisone was increased to more than 30 mg/day. He presented with symptoms of itchy skin and rash of limbs, and was diagnosed with “allergic dermatitis” in October 2012. Swelling of neck lymph node was first noted in 2013. Ultrasound detected found that the largest lymph node in the left neck was 1.6 **×** 0.7 cm. He rejected lymph node biopsy. Prednisone was reduced and stopped gradually, proteinuria remained negative since 2014.

On admission, blood pressure was 151/92 mmHg. Enlarged lymph nodes with clear boundary and good range of motion was noted in the neck, supraclavicular, and inguinal region. The mass of right retroauricular was circular, soft, no-tender with a moderate range of motion, and measured about 2 cm in diameter. The WBC was 24.54 **×** 10^9^/L, with 20.4% neutrophils, 9.3% lymphocyte and 67.3% eosinophils. Urinary red blood cell (RBC) count was 760**,**500 cells/ml with mixed type of predominant dysmorphic RBC. 24-h urinary protein was 0.36 g and serum albumin was 43.8 g/L. The serum creatinine level and estimated glomerular filtration rate (eGFR) were 88 μmol/l and 90.99 mL/min per 1.73 m^2^, respectively. The serum IgE level increased to 1735 KU/L (reference value: **<** 60 KU/L). Sensitive troponin I was elevated to 0.0907 ng/ml. There were no abnormalities in liver function, coagulation function and urine culture. No evidence of infectious disease, connective tissue disease, and malignant tumor was found in the laboratory examinations. Ultrasound detected no abnormalities in urinary system, while lymph nodes enlargement was found in the neck, supraclavicular, subclavian and inguinal regions. The largest lymph nodes were 1.4 **×** 1.0 cm in the neck, 1.8 **×** 1.1 cm in left subclavian, and 2.0 **×** 0.8 cm in inguinal region, respectively. Computed tomography (CT) showed multiple enlarged lymph nodes in mediastinum, bilateral axillary and retroperitoneum. Electrocardiogram revealed left ventricular hypertrophy with strain and unusual Q waves in V2-V3. Echocardiography showed left ventricular dilation and dysfunction, and significantly decreased systolic activity of the inferior wall of left ventricle. Left ventricular ejection fraction (EF) was 30%. Coronary CT angiography (CTA) did not detect significant stenosis of each branch.

**Fig. 1:**
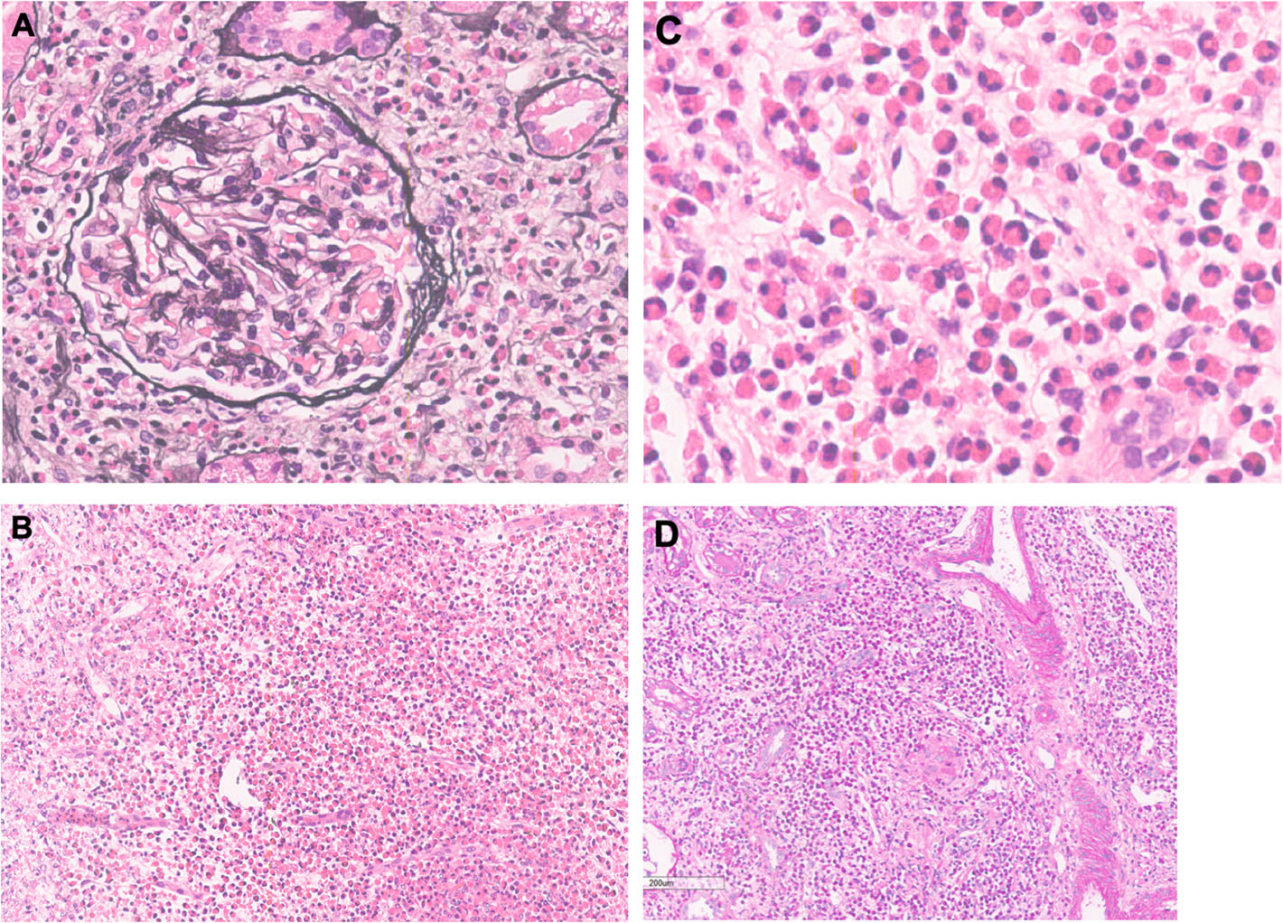
Light microscopy of samples from renal biopsy. Only a glomerulus showed fibrocellular crescent (1/26) (**A**: PASM). Multifocal and patchy eosinophil infiltration in the interstitium was noted (**B**, **C**: PAS). The renal tubules showed unclearly because of massive eosinophil infiltration (**D**: PAS). (PASM, periodic acid-silver methenamine; PAS, periodic acid-schiff)

**Fig. 2:**
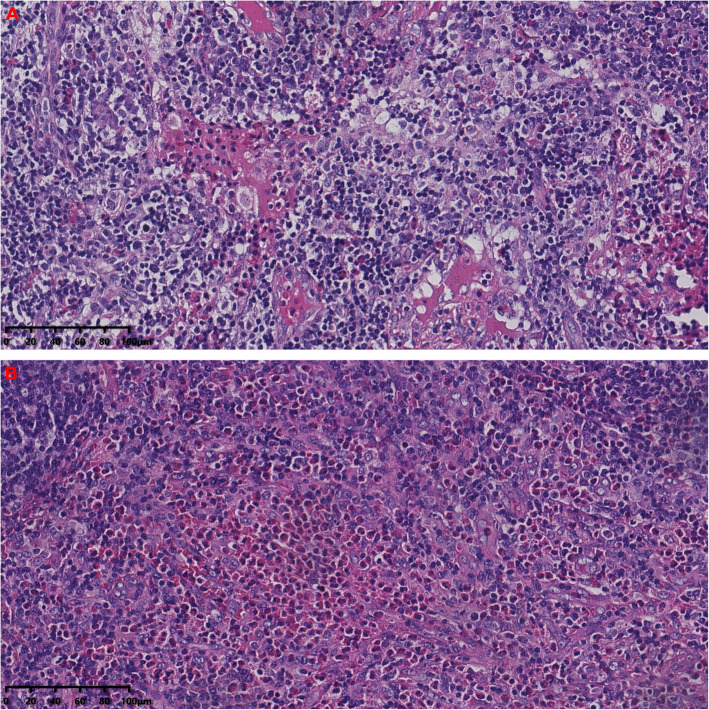
Lymph node pathology features. The Acidophilic granulocyte infiltrated into the lymph node (**A**: HE). The structure of lymph node was destroyed by acidophilic granulocyte (**B**: HE). (HE, hematoxylin and eosin)

To identify the cause of eosinophilia, bone marrow puncture and renal biopsy were performed. Bone marrow cell morphology indicated eosinophilia with 9% myelocyte, 1% metamyelocyte, 8% stab granulocyte, and 20% segmented granulocyte. Eosinophilic interstitial nephritis was diagnosed by renal biopsy (Fig. 1). Light microscopy showed multifocal and patchy infiltration of eosinophils (about 70% area) in interstitium, granular degradation and tubular vacuolization of renal tubular epithelial cells, thickening and narrowing of arteriolar wall, while no significant proliferation of mesangial cells and matrix or thickening basement membrane was observed. Immunofluorescence of IgG, IgA, IgM, C3, C1q, Fib, IgG1, IgG4, and phospholipase A2 receptor (PLA2R) were negative. Electron microscope showed diffuse foot process fusion and only a few electron dense deposits in the mesangium region. Based on results from cervical lymph node biopsy that histological examinations showed eosinophil hyperplasia, destruction of germinal centers by eosinophils (Fig. 2), final diagnosis of KD was made.

**Fig. 3:**
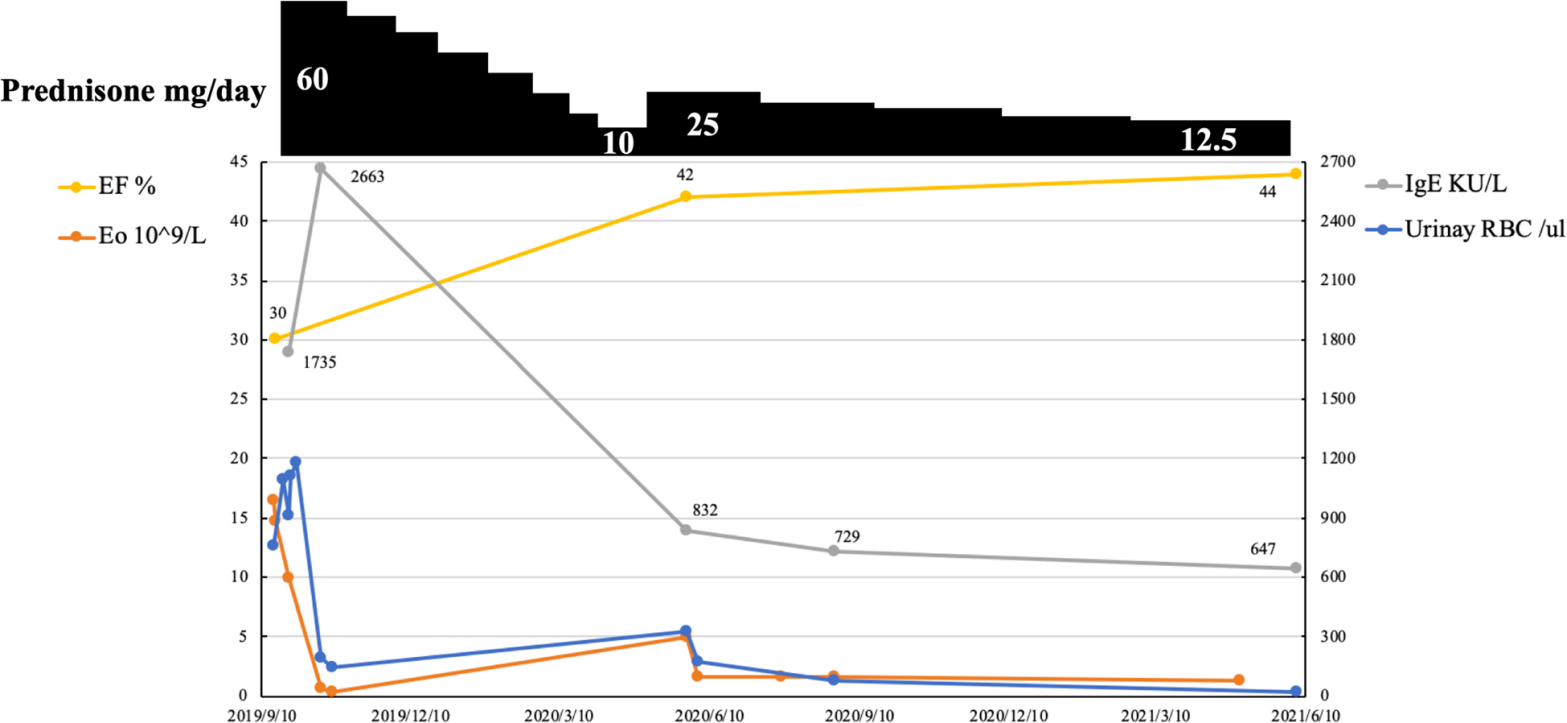
Clinical course. (Eo: eosinophils, EF: ejection fraction, Urinary RBC: urinary red blood cell count, IgE: immunoglobulin E)

The patient was treated with oral prednisone therapy (60 mg/day) on October 10, 2019. Because of 2019 novel coronavirus, the patient didn’t return visit. After 7 months of treatment with prednisone, the subcutaneous masses in forehead and right retroauricular regressed. Meanwhile, eosinophil count, serum IgE, and urinary RBC count decreased. However, hematuria and eosinophilia recurred when prednisone gradually reduced to 10 mg/day. The patient was given prednisone at 25 mg/day on May, 2020 and decreased to a maintenance dose of 12.5 mg/day. Eosinophil count, urinary RBC count, and IgE reduced, while EF increased to 44% (Fig**.** 3). He kept normal renal function (serum creatinine 88 μmol/l, eGFR 90.35 mL/min per 1.73 m^**2**^) during follow-up.

## Discussion and conclusions

We present a case of nephrotic syndrome earlier than subcutaneous masses, along with remarkable eosinophilia, elevated serum IgE, multiple lymphadenopathy and cardiac insufficient. KD should be differentiated from other diseases such as Hodgkin’s disease, metastatic nodules, parasitic diseases, allergic diseases, cervical lymph node tuberculosis, eosinophilic leukemia, IgG4 related disease. In this case, the pathology of the lymph node plays a definitive role in the diagnosis of KD. Histopathology shows extensive formation of lymphoid follicles and a large number of eosinophils, lymphocytes and mast cells between follicles, and eosinophilic granulomas are common [6]. Angiolymphoid hyperplasia with eosinophilia (ALHE) is easy to be confused with KD. The identification between the two mainly depends on histology [6]. Renal damage is more common in KD than ALHE [7]. KD also needs to be differentiated from the idiopathic hypereosinophilic syndrome (IHES). IHES can involve multiple system, approximately 60% with cardiac damage [8], while renal involvement is rare.

Most renal damage appeared months or years after subcutaneous mass or lymphadenopathy [9–11], while some were found simultaneously [12], and rarely preceded the presence of masses [3, 13]. In this patient, the nephrotic syndrome and eosinophilia predated the discovery of cervical lymphadenopathy by 3 years, we speculated that the use of glucocorticoid suppressed the formation of the angiolymphoid masses and delayed the diagnosis of KD. Various types of glomerulonephritis are associated with KD. However, it has been rarely reported that renal injury in KD showed obvious interstitial inflammatory cell infiltration and mild tubulopathy, while only a few reports mentioned glomerular lesion with interstitial eosinophilic infiltration [14]. Yang S et al. [14] retrospectively reviewed 12 cases of KD with kidney involvement. Four cases showed concurrent minimal change disease (MCD) and interstitial lesions. They concluded 2 distinct patterns: exclusively eosinophilic infiltration or diffusely eosinophilic and lymphatic infiltration in the interstitium similar to KD lesion. The clinical manifestations of our patient were characterized by massive proteinuria and hematuria, respectively, while renal biopsy showed alone marked interstitial lesion. The etiology and pathogenesis of KD and renal damage are still unclear. It has been considered that cytokines released by activated T cells contribute to eosinophil proliferation, serum IgE elevation, the progression of lymph node changes and renal damage, as well as increasing the permeability of the glomerular basement membrane [3, 10].

There are only few reports on KD with cardiac damage. Horigome et al. [5] reported a 13-year-old boy diagnosed with KD according to upper arm nodule biopsy. He showed recurrent chest pain, syncope, and even ventricular fibrillation, confirmed to have coronary vasospasm without obvious atherosclerosis or thrombosis, which could not be relieved by nifedipine and isosorbide, but was inhibited by prednisolone. The pathogenesis of KD complicated with cardiac damage is not clear. Spry et al. [15] demonstrated that the serum eosinophil granule protein, especially cationic protein, which was higher in KD patients than healthy people. Eosinophil granule proteins could produce muscle damage and vascular injury which lead to the development of endomyocardial fibrosis. Reports of both cardiac and renal damage in KD are even rarer. Mahapatra et al. [16] reported a KD case in a 35-year-old Indian male presenting simultaneously with diffuse proliferative glomerulonephritis and complete heart block. To the best of our knowledge, we firstly reported a KD patient with both independent renal interstitial and unexplained cardiac insufficiency. Our patient had no past history of heart disease. CTA found no organic stenotic lesions, echocardiography revealed decreased left ventricular function. After prednisone treatment, the subcutaneous mass shrank, eosinophils and blood IgE decreased while the left ventricular function increased significantly. Consistent with the previous report [5], KD with cardiac damage was sensitive to glucocorticoid therapy. If further endomyocardial biopsy can be performed, it will be of definite value in the diagnosis.

An optimal therapy for KD is not defined due to the rarity of the disease, misdiagnosis and loss of follow-up. It is generally believed that KD-associated renal lesion is a benign course, only a few patients have been reported to progress to end-stage renal disease requiring dialysis [3, 17]. Glucocorticoid is the first choice for medical treatment of KD with nephropathy [11], but it is easy to recur after glucocorticoids withdrawal or dose reduction [3, 9]. Although this patient had multiple relapses over a decade, he had a good response to glucocorticoid and required a small effective dose (≤25 mg/d) without deterioration of renal function. The remission time was shorter than that of steroid-sensitive primary nephropathy. Immunosuppressive agents such as cyclosporine, cyclophosphamide, vincristine, tacrolimus, leflunomide, tripterygium wilfordii, and myophenolate mofetil have also been reported to be effective in the treatment of KD with renal damage or can reduce the recurrence caused by glucocorticoids dose reduction [4, 12, 13]. Surgical excision is the most widely used treatment for local mass. Lee et al. [9] reported that elevated serum creatinine returned to normal and proteinuria was relieved within 1 month of resection of the subclavian mass in a KD patient with suboptimal response to prednisolone and cyclosporine. Radiotherapy can be another effective option for steroid-resistant KD patients. Chang et al. [18] concluded that low-dose radiotherapy (>25Gy) was superior to surgical resection or steroid therapy. Although KD is considered to be a benign progressive disease, its recurrence rate can be 17–44% [19]. Some scholars have proposed that peripheral eosinophils over 20%, serum IgE levels more than 10,000 IU/ml, multifocal lesions outside the salivary glands, disease duration more than 5 years, bilateral involvement, a lesion diameter of greater than 3 cm, and ill-defined lesions are prognostic factors for disease recurrence [19, 20]. Our patient had at least two risk factors and needs further follow-up to observe the prognosis.

In summary, we report a KD case with renal involvement 3 years earlier than subcutaneous masses. He presented with eosinophilic interstitial nephritis, cardiac insufficiency and significant response to prednisone. The clinicians should be vigilant for the occurrence of KD, if the patient shows steroid-sensitive renal damage combined with peripheral eosinophilia and increased IgE, even without subcutaneous mass or lymphadenopathy. More researches on the pathogenesis of KD are needed to provide immunological treatment basis.

## Data Availability

All data related to this case report are within the manuscript.

## Abbreviations

KD: Kimura’s disease
WBC: White blood cell
RBC: Red blood cell
eGFR: Estimated glomerular filtration rate
CT: Computed tomography
EF: Ejection fraction
CTA: Coronary computed tomography angiography
ALHE: Angiolymphoid hyperplasia with eosinophilia
IHES: Idiopathic hypereosinophilic syndrome
MCD: Minimal change disease
IgE: Immunoglobulin E.
